# Healthcare Utilization and Smoking among South Carolina’s Long-Term Uninsured

**DOI:** 10.3390/healthcare10061079

**Published:** 2022-06-10

**Authors:** Caitlin Torrence, Khoa Truong, Laksika B. M. Sivaraj

**Affiliations:** 1 Department of Public Health Sciences, Clemson University, Clemson, SC 29634, USA; ctorren@clemson.edu (C.T.); lsivara@clemson.edu (L.B.M.S.); 2 Office of Research and Organizational Development, Clemson University, Clemson, SC 29634, USA

**Keywords:** smoking, tobacco, uninsured, insurance, health disparities

## Abstract

Cigarette smoking and tobacco-related health conditions have continued to rise among persons of low social economic status. This study explored the association between healthcare utilization and smoking among the long-term uninsured (LTU). The sample consisted of South Carolina residents who had been without healthcare insurance for at least 24 months. Multivariable logistic regression was used to estimate differences in the likelihood of delaying healthcare due to cost and/or not filling a needed prescription between smokers and non-smokers. Among LTU, smoking was a significant predictor of delaying healthcare at the 10% level (AOR = 1.36, 95% CI = 0.99–1.86); the sensitivity analysis strengthened this association at the 5% level (AOR = 1.43, 95% CI = 1.06–1.93). Smoking was a significant predictor of not filling needed prescriptions (AOR = 1.44, 95% CI = 1.06–1.96). While neglected healthcare utilization was common among the LTU, this problem was more severe among smokers. The wider gap in access to healthcare services among the LTU, especially LTU who smoke, warrants further attention from the research community and policy makers.

## 1. Introduction

Through the Affordable Care Act (ACA), the government tries to improve access and quality of care for disadvantaged groups. Even though the ACA has been successful in providing health care coverage to many low-income individuals through Medicaid, there are still a considerable number of individuals left uninsured. This is often because they earn just above the Medicaid threshold for eligibility, but they do not earn enough to afford private insurance.

Health behaviors differ between the uninsured and the insured. Persons of lower socioeconomic status (SES) are more likely to engage in negative health behaviors [1,2,3,4,5,6,7,8]. For instance, in 2019, the prevalence of tobacco use was 18% for privately insured adults but a staggering 30.2% for uninsured adults. The United States (US) government has reduced cigarette consumption among persons of middle and high SESs, yet nicotine consumption has continued to rise among persons of low SES [9,10].

Unfortunately, health-seeking behaviors often compound this scenario. The uninsured are more likely to delay care and have a harder time finding providers. People who delay health care are sicker and require more treatment when they do access healthcare. Given that in 2017 alone, USD 33.6 billion public funds were used to offset uncompensated care cost for the uninsured, this has become a national concern [11]. This situation is projected to become more serious as the uninsured US population was expected to grow by 1.5 million between 2017 and 2019 [11].

This volatile marriage between health behaviors and health care utilization among the uninsured is especially pronounced in the context of smoking. A recent systematic review of inequalities related to smoking found that among the many influences of the socioeconomic gradient in health, smoking consistently contributed the most to all-cause mortality [1] and other smoking-related health disparities [2,5,6,12]. For instance, several studies have documented that smoking results in higher tobacco-related illnesses and mortality rates among the poor [1,2,6,9,13,14,15].

While the body of knowledge on smoking is vast, understanding of the intersection between smoking and health care utilization of special population subgroups such as the long-term uninsured is limited [10]. Given that most of the uninsured are of low SES and this economic bracket has a high prevalence of tobacco use, exploring the effect of smoking on healthcare utilization among the long-term uninsured (LTU) could bring a new perspective to the literature [8,16]. This study investigated potential differences among the health-seeking behaviors of the uninsured. It was hypothesized that among the long-term uninsured, compared to non-smokers, smokers would be more likely to delay health care when needed and less likely to fill prescriptions when needed. The underlying premise is that the cost to sustain the smoking behavior takes away limited financial resources that would otherwise be spent on health care when needed.

## 2. Materials and Methods

### 2.1. Data

Data for this study were collected from May 2014 to January 2015 in South Carolina using a multi-stage sampling method. South Carolina residents aged 18–64 who were LTU at the time of the survey were eligible to participate. LTU was defined as having been without health insurance for 24 months or longer. The LTU study was administered through an in-person interview. Interview questions focused on smoking status, health status, access to healthcare services, health service utilization, and attempts to gain health coverage. There were 954 completed interviews. Details of the interview questions, including sampling method and data collection, are detailed elsewhere [17]. After excluding 5 participants due to missing responses, the final analytic sample included 949 respondents. The study protocol and methods were approved by the Institutional Review Board of Clemson University, who also determined that the use of data in this manuscript does not involve human subjects and is thus not subject to IRB review.

### 2.2. Dependent Variables

Three outcomes were examined in this study. Each outcome was constructed as a binary variable. The first measure was delaying healthcare when needed due to cost. Participants were asked, “Was there a time in the past 12 months when you needed to see a doctor but could not because of cost?” Those answering “Yes” were coded as a “1”, otherwise “0”. The second measure was being unable to fill a needed prescription. Participants were asked, “Was there a time in the past 12 months when you needed to get a prescription but could not?” Coding was the same as the first measure. These two measures were then combined to construct the third outcome variable. If the response to either or both questions was a “Yes”, the third measure was coded as “1”, otherwise it was coded as “0”.

### 2.3. Independent Variables

The key independent variable was smoking status as a regular smoker. Respondents were asked, “Do you regularly smoke cigarettes, cigarillos, cigars, or use any kind of tobacco products?” Respondents who answered affirmatively were classified as smokers. Guided by the previous literature [18,19] and the available data from the LTU survey, selected demographic characteristics included age, gender, race, marital status, educational achievement, employment status, household size, and household income. Age (in years) and household size (number of members) were continuous variables. Race was originally five categories; however, due to very few respondents in the Asian (0.2%), Indian/Native American/Alaskan Native (2.1%), and some other race categories (8%), race was collapsed into three categorical variables (African American, White, and all other races). Marital status was dichotomized by collapsing the categories married, common-law married, and living with partner into “married/living as married” and by collapsing the categories divorced, separated, never married, and widowed into “unmarried”. The education levels reported in the survey ranged from no formal education to advanced degrees. For this analysis, education was dichotomized into less than high school diploma and high school diploma or greater. Annual household income was modeled as categorical variables by dividing the respondents into the 5 income quintiles (20%, 40%, 60%, 80%, and 100%).

To add additional perspective to the primary health-related outcomes, three health-related characteristics were also included as covariates. Participants were asked to rate their health using the General Self-Rated Health question, “Would you say your health is: excellent, very good, good, fair, or poor.” This is a reliable subjective measure of health status [20]. For analysis purposes, due to the small percentages at either ends of the response scale (excellent, 10%; poor, 9%), the variable was collapsed into three categories: (1) fair or poor health, (2) good health, and (3) very good or excellent health. Participants were asked about the last time they visited a doctor for a routine check-up. They were provided with the following options: “within the past year”, “within the past 2 years”, “within the past 5 years”, “5 or more years ago”, “don’t know/not sure”, and “never”. As it is recommended to have a wellness check-up annually and due to the 12-month timeframe of the outcome variables, this measure was dichotomized to be interpreted as “had a wellness check-up within the past year” or “did not have a wellness checkup within the past year”. Finally, respondents were asked, “Have you ever been told that you have any serious long-lasting health problem such as diabetes/high blood sugar, high blood pressure, or high cholesterol?” This question helps establish a baseline of health for respondents and potentially indicates a sustained need for healthcare interactions. Responses were documented as yes or no.

### 2.4. Statistical Analysis

Descriptive statistics for demographic and health-related characteristics were provided for the entire sample and by smoking status. To compare the groups, Pearson chi-squared tests were used for categorical variables, and t-tests were used for continuous variables. Multivariable logistic regression models were used to analyze each of the three binary dependent variables. Each logistic regression model included the same set of covariates described above. Covariate selection was based on the literature [1,2,3,21] and supported by using the change-in-estimates method to build the model. The change-in-estimates method involves starting with the crude model and adding one variable at a time. If the coefficient of the key independent variable increases by more than 30 percent when a covariate is added, the added variable is considered a confounding variable and is excluded from the full model. All models were assessed for goodness-of-fit using the linktest and the lfit group (10) Stata command, and multicollinearity was assessed using the variance inflation factor (vif) command. Each model was correctly specified and had an acceptable mean vif. Statistical analyses were conducted using Stata v. 14.0. For all models, adjusted odds ratios (AORs) and 95% confidence intervals (CI) were reported. To provide an easier and meaningful interpretation of the AORs from the multivariable logistic regression models, the differences in percentage of each outcome by smoking status were estimated. The estimation was executed through the following steps: First, after the logistic regression for each outcome was run, the “predict” command was used in Stata to estimate the adjusted probability of the outcome for each respondent. Next, the average adjusted probability was calculated for smokers and non-smokers. Lastly, the difference in the average adjusted probabilities between the two groups (smokers vs. non-smokers) was computed.

In order to perform the sensitivity analysis, the first outcome (delayed healthcare due to cost) measure was refined using another interview question, “What is your ‘wish list’ to improve services from the care providers you have received care?” Choice options included: (1) “Their service should be near where you live”, (2) “They should be more willing to take your insurance”, (3) “They should charge you less for the co-pay or co-insurance”, (4) “They should offer more service hours (i.e., they should be “open” more often)”, (5) “They should have more types of services available”, (6) “There should be less paperwork burden”, (7) “Cost should be lower”, (8) “They need more understandable and friendly communication from the healthcare providers”, and (9) “Other”. Multiple answers were allowed. The sensitivity analysis of model one converted participants who reported that they had delayed care due to cost within the past 12 months but did not report lower cost as a “wish list” item from affirmative to negative responses. The rationale for this analysis rested on cost being identified as a significant barrier when seeking healthcare. If cost did serve as a significant barrier, then cost should, theoretically, also be listed as a wish list item.

## 3. Results

Table 1 shows the descriptive statistics of the analytic sample. Of the total 949 respondents, just under half (44.9%) reported smoking regularly. The average age of respondents was 42 (SD = 12.7). Of all respondents, 58.2% were female. The racial make-up of the sample was primarily African American (72%), then White (17.6%), and other races (10.4%). Most of the respondents reported not being married (84.2%). The education level of respondents tended to be low. Over half (53.6%) reported having less than a high school diploma. A majority of respondents (73.5%) reported not working. On average, the household size for respondents was 2.5 (SD = 1.5). As to overall health status, 42.2% reported being in poor or fair health, 30.8% reported being in good health, and 27% reported being in very good or excellent health. Just over 55% of respondents reported having a chronic health problem, and 62.4% of respondents indicated that they had not had a routine check-up during the past 12 months. As many as 41.3% of respondents reported that they had at some point in the past received advice or guidance on smoking cessation from a healthcare provider.

More than half of respondents (n = 602, 63.2%) reported that they had delayed seeking healthcare within the past 12 months due to cost. Among these respondents, there were no statistically significant differences in the percentage between smokers and non-smokers. There were, however, significant differences of delayed healthcare among racial identity (*p* = 0.009), working status (*p* = 0.041), self-reported health (*p* < 0.001), and routine check-up (*p* < 0.001). African Americans and those not working were the most likely to delay healthcare due to cost. Those who reported poor or fair health were also more likely to delay healthcare due to cost.

Over half of the respondents (*n* = 498, 52.5%) reported that they had been in a situation within the past 12 months when they were unable to obtain a needed prescription. Among these respondents, there was a significant difference in smoking status (*p* = 0.023). Smokers were more likely to report that they could not obtain a needed prescription. There were also significant differences in the proportion of respondents who could not obtain a prescription across gender (*p* = 0.002), marital status (*p* = 0.013), working status (*p* = 0.04), self-reported health (*p* < 0.001), chronic health problem (*p* < 0.001), and healthcare smoking cessation support (*p* = 0.001). Women and those not married, not working, in poor or fair health, who had a chronic health problem, and who had received smoking cessation support in the past were more likely to report that there was a time in the past twelve months in which they could not obtain a needed prescription.

Those reporting that they had delayed care due to cost and/or could not obtain a needed prescription within the past 12 months made up the majority of the sample (*n* = 689, 72.5%). There was a significant difference at the 10% level in the proportion of these respondents across smoking status (*p* = 0.06), but women (*p* < 0.001), African-American (*p* = 0.024), not working (*p* = 0.034), poor or fair health (*p* < 0.001), having a chronic health condition (*p* < 0.000), not having routine checkup (*p* = 0.015), and having received smoking cessation support (*p* = 0.012) were more likely to delay healthcare and/or not obtain a needed prescription.

Table 2 shows the results of the multivariable logistic regression for all three outcomes. The logistic regression model for the first outcome (delaying healthcare) was statistically significant; X2 (17, *n* = 826) = 69.56, *p* < 0.001. The model explained 6% of the variance in delaying healthcare due to cost. While there was a significant difference at the 10% level, the 5% level did not result in a statistically significant difference between smokers and non-smokers who delayed healthcare due to cost (AOR = 1.36, 95% CI = 0.99–1.86). The logistic regression model for the second outcome (filling prescriptions) was statistically significant; X2 (17, *n* = 822) = 78.76, *p* < 0.001. The model explained 7% of the variance in not filling prescriptions when needed. Smoking was significantly associated with not obtaining a needed prescription (AOR = 1.44, 95% CI = 1.06–1.96). The logistic regression model for the third outcome (delaying healthcare and/or filling prescriptions) was statistically significant; X2 (17, *n* = 823) = 78.90, *p* < 0.001. The model explained 8% of the variance in delaying healthcare due to cost and not filling needed prescriptions. The combination of these two outcomes was statistically significant (AOR = 1.59, 95% CI = 1.11–2.26). For all models, compared with females, the AOR was lower for males, and compared with those in poor or fair health, the AOR was lower for those reporting good, very good, or excellent health.

The sensitivity analysis using the “wish list” to redefine the first outcome, delayed healthcare due to cost, converted 120 responses from affirmative to negative responses. After running the multivariate logistic regression model with this adjustment to the first outcome variable, smokers were significantly more likely than non-smokers to report delaying healthcare due to cost (AOR = 1.43, 95% CI = 1.06–1.93).

Table 3 presents the simulated outcomes. Among the LTU, the percentage of those delaying healthcare due to cost was 68% (95% CI: 66–69%) among smokers and 62% (95% CI: 60–63%) among non-smokers. Significant results were also found for the second outcome. The percentage of those reporting not filling a needed prescription during the past 12 months was 59% (95% CI: 57–60%) among smokers and 50% among non-smokers (95% CI: 49–52%). Combining these two outcomes together, 78% (95% CI: 77–79%) of smokers either delayed healthcare due to cost or could not obtain a needed prescription, compared with 70% (95% CI: 69–71%) of non-smokers.

## 4. Discussion

This study extends the analysis on the socioeconomic gradient in health to the LTU, a unique sub-group within America’s low-income population who have been without health insurance for 24 months or longer. It re-affirms previous findings whereby persons of lower income are more likely to smoke in the US [9,10,18,19] and indicates that there are differences in smoking status by lower income subgroups, such as the LTU. For instance, while 20% of South Carolina residents report smoking [22,23], this percentage increases among participants in the LTU study. Nearly half (44%) of all participants reported smoking regularly at the time of the interview. Smoking is known to cause poor health outcomes, and there is a similar association between lack of insurance and health outcomes.

Many of the same risk factors for smoking are associated with being uninsured [18], which warrants examination of the influence of smoking on the socioeconomic gradient in health among the uninsured [18,24]. In the US, the working poor, males, and persons with lower educational attainment are over-represented among the uninsured population [18,19]. While males are over-represented among the LTU in general, this study found that females are more likely to delay seeking needed healthcare and are unable to obtain needed prescriptions. It suggests that this is amplified among smokers. It is counter-intuitive, because compared to males, females are generally more proactive in seeking healthcare. Perhaps females among the LTU are more likely to neglect healthcare needs if they are primary caregivers for children. It is also possible that females have more healthcare needs than men resulting in less accessibility to healthcare services due to high cost. While a large percentage of our sample reported not currently working, in general, the LTU are likely among the working poor. Their income is too high to qualify for Medicaid but not enough to cover their bills or pay for insurance [18,24]. This could also limit the resources of women with children.

Furthermore, the data were collected between 2014 and 2015, prior to the COVID-19 pandemic. The working poor and the uninsured—those most likely to be represented in this study’s sample—have been disproportionately affected by the pandemic. Not only were the economic livelihood of shift workers most negatively affected by national precautions to stem the spread of the virus, such as stay-at-home orders, but negative health behaviors including nicotine use have increased. While Knell et al. found that in the early days of the pandemic, health behaviors mostly stayed the same, women, those with children, and persons with higher depressive scores were more likely to increase negative health behaviors, including smoking [25]. Unfortunately, smoking and other negative addictive behaviors are not easily changed as society adapts to the “new normal.” Therefore, there is a heightened necessity to understand the unique healthcare needs and behaviors of the LTU.

While this study was unable to determine the underlying reasons and motivations that influence how cost factors into a person’s assessment of seeking healthcare and obtaining necessary prescriptions, the cost of buying cigarettes could be partially responsible for more neglected healthcare utilization among smokers. More research is, therefore, needed to investigate this type of potential adverse effect of smoking on healthcare usage behavior and its downward effect on health. It is possible that tobacco control policies can play a role, either in a positive or negative way, in the conscious and subconscious decision-making processes regarding utilizing healthcare. For instance, raising excise tax on cigarettes discourages consumption generally (positive effect) but imposes further financial burden on the low-income uninsured smokers, resulting in the unintended negative consequence of trading off healthcare for cigarettes.

It was found that smoking status among the LTU results in different experiences accessing and utilizing healthcare as well as complying with medical guidance such as filling prescriptions. While delaying healthcare is common among the LTU, smokers are more likely to delay healthcare when needed due to cost and are significantly more likely to be unable to obtain a needed prescription. This is concerning, because studies consistently find that health care providers who offer aid in the form of advice, counseling, and prescriptions are important for successful smoking cessation [10,15,26]. Smokers who receive healthcare provider assistance including prescription aids for smoking cessation in addition to smoking cessation counseling have the greatest success in quitting [10]. In fact, this study supports that the physicians follow these guidelines. Over half (64.2%) of smokers who reported seeking healthcare at some point in the past reported that his or her doctor had provided smoking-control advice, education, or support. However, when healthcare providers follow care guidelines and prescribe medication, among the LTU, smokers are less likely to follow through. This is especially alarming when accounting for the over-representation of smoking-related negative health consequences among low-income persons [2,5,6,12]. The LTU included in our sample were notably poor, yet smokers were most negatively affected by cost.

The limitations of our study should be recognized. First, our survey relied on self-reports that can result in under-reporting of undesirable behaviors or outcomes. However, if the level of under-reporting was similar between smokers and non-smokers, our estimated coefficients would not be biased for this specific reason. Further, high validity with reporting smoking status has been found in surveys [26]. Second, participants were asked if they regularly smoked or used tobacco products of any kind. Specific forms of smoking, beyond those which were referenced in the survey question, were not asked of participants. Frequency of smoking or tobacco use was not further defined within the survey and interpretation resided with the participant. Third, it was not possible to determine the causal effect of smoking on healthcare utilization due to the cross-sectional nature of our survey. Fourth, compared with non-smokers, smokers may be more (or less) likely to have unobserved characteristics that are correlated with neglected healthcare utilization, thus biasing the estimation of the key independent variable.

## 5. Conclusions

South Carolina, a non-Medicaid expansion state with a high uninsured rate, has many residents who are among this unique subgroup. South Carolina’s uninsured rate in 2013 was 15.8%, compared with the national uninsured rate of 14.5% for states that did not accept the Medicaid expansion [27]. The Kaiser Family Foundation estimated that in 2014, the year in which the mandate went into effect, if South Carolina had expanded Medicaid, approximately 20% of people without insurance would have received coverage [28]. By 2018, the uninsured rate in South Carolina remained 2.3% higher than the national rate [29]. In addition to high uninsured rates, South Carolina also has high smoking rates compared with the national average. The findings of this study, therefore, have important implications for efforts to improve healthcare access for disadvantaged population, including the smoking LTU.

Many studies have explored the relationship between income status and healthcare utilization. They have consistently found that low SES is associated with poor health outcomes that are often attributed to smoking [1,21,30,31]. This study supports previous findings and extends the conversation on how smoking influences the socioeconomic gradients in health with a focus on the LTU. In general, the LTU are poor, have limited access to healthcare, and face substantial barriers to receiving medical attention. Among this population, smokers are significantly more likely to delay healthcare when needed and not fill needed prescriptions. Cost was the most reported barrier among LTU smokers, which, perhaps, offers some explanation about reduced healthcare utilization. These findings warrant further research to explore the influence of smoking on health disparities within unique subgroups of the American low-income population and to devise strategies for addressing health care needs and smoking cessation.

Future studies should further explore the relationship between the LTU and smoking, as it relates to health disparities. For example, a better understanding of the unique social norms relative to and motivating factors for smoking and seeking healthcare among the LTU could support the development of smoking cessation interventions and healthcare programming that are tailored to the specific needs of this low-SES subgroup. Additional research should examine the types of prescriptions among this population that have the greatest likelihood of not being filled. This would help to build applied knowledge that could be practically utilized in healthcare settings. For example, if the smoking LTU are more likely to report being unable to fill a smoking cessation prescription due to cost, then effort could be made to provide samples of cessation medications to patients who are chronically uninsured.

## Figures and Tables

**Table 1:** Descriptive statistics of the study sample (*n* = 949).

Variables	Response/Self-Report	^+^ Delayed Healthcare due to Cost	^+^ Did not Fill Prescription	^+^ Delay Cost and/or did not Fill Prescription
	*n* (%)	*n* (%)	*n* (%)	*n* (%)
*Self-Reported (Yes)*		602 (63.2) ^1^	498 (52.5) ^2^	689 (72.5) ^3^
*Regular smoker*			*	
Yes	426 (44.9)	278 (46.3)	239 (48.1)	320 (46.5)
No	523 (55.1)	323 (53.7)	258 (51.9)	368 (53.5)
*Age*				
Mean (SD)	42.0 (12.7)	42.1 (12.1)	42 (12.1)	42.0 (12.2)
*Gender*			**	***
Male	396 (41.8)	219 (36.5)	183 (36.9)	259 (37.7)
Female	551 (58.2)	381 (63.5)	313 (63.1)	428 (62.3)
*Race*		**		**
White	167 (17.6)	116 (19.3)	92 (18.5)	132 (19.2)
African American	682 (72.0)	412 (68.7)	356 (71.6)	478 (69.6)
Other	98 (10.4)	72 (12	49 (9.9)	77 (11.2)
*Marital Status*			*	***
Married ^a^	150 (15.8)	97 (16.1)	65 (13.1)	104 (15.1)
Not married	799 (84.2)	504 (83.9)	432 (86.9)	584 (84.9)
*Education*				
<High school	499 (53.6)	309 (52.7)	251 (51.3)	353 (52.5)
≥High school	432 (46.4)	277 (47.3)	238 (48.7)	320 (47.5)
*Working*		*	**	*
Yes	251 (26.5)	146 (24.3)	112 (52.6)	170 (24.8)
No	695 (73.5)	454 (75.7)	383 (77.4)	516 (75.2)
*Household size*				
Mean (SD)	2.5 (1.5)	2.5 (1.6)	2.5 (1.5)	2.5 (1.5)
*Self-reported health*		***	***	***
Poor/fair	401 (42.2)	293 (48.7)	258 (51.9)	332 (48.3)
Good	292 (30.8)	179 (29.8)	142 (28.6)	206 (29.9)
Great/excellent	256 (27.0)	129 (21.5)	97 (19.5)	150 (21.8)
*Chronic health problem*		*	***	***
Yes	424 (44.7)	287 (47.8)	263 (53.1)	334 (48.7)
No	524 (55.3)	313 (52.2)	232 (46.9)	352 (51.3)
*Routine checkup (past 12 months)*		**		**
Yes	348 (37.6)	287 (47.8)	177 (36.3)	236 (35.1)
No	578 (62.4)	313 (52.2)	311 (63.7)	437 (64.9)
*Healthcare smoking cessation support*			***	*
Yes	390 (41.3)	257 (43.0)	229 (46.4)	299 (43.7)
No	555 (58.7)	341 (57.0)	265 (53.6)	385 (56.3)

+ Each outcome (delayed healthcare due to cost, did not fill prescriptions, and delayed healthcare due to cost and/or did not fill prescription) was modeled individually. * *p* ≤ 0.05; ** *p* ≤ 0.01; *** *p* ≤ 0.001. When there are significant differences between how respondents answered a demographic question and the outcome variable (table column), the significance indicator is provided on the row listing the demographic characteristic in the corresponding outcome variable column. ^a^ Married or living as married; ^1^ 1 missing value; ^2^ 5 missing values; ^3^ 4 missing values. Gray highlighted indicate the descriptive statistics for the overall study sample. Italic indicates the row headings.

**Table 2:** Association between smoking and key outcomes variables ^+^.

Covariates ^a,b^	^+^ Delayed Healthcare due to Cost ^c^ AOR (95% CI)	^+^ Did not Fill Prescription ^c^ AOR (95% CI)	^+^ Delay Cost and/or Did Not Fill Prescription ^c^ AOR (95% CI)
Smoking	1.36 (0.99–1.86)	1.44 (1.06–1.96) **	1.59 (1.11–2.26) **
Age	0.99 (0.98*–*1.00)	0.99 (0.97*–*0.99) *	0.99 (0.98*–*1.00)
Male	0.57 (0.41*–*0.78) ***	0.69 (0.50*–*0.94) *	0.55 (0.39*–*0.78) ***
African American	0.66 (0.43*–*1.02)	1.00 (0.67*–*1.50)	0.65 (0.40*–*1.05)
Other race	0.96 (0.49*–*1.88)	1.09 (0.58*–*2.03)	0.88 (0.42*–*1.85)
Married	0.88 (0.57*–*1.37)	0.63 (0.41*–*0.97) *	0.64 (0.40*–*1.02)
≥High school graduate	1.02 (0.75–1.38)	1.09 (0.82*–*1.47)	1.10 (0.79*–*1.54)
Working	0.84 (0.60*–*1.19)	0.78 (0.55*–*1.09)	0.87 (0.60*–*1.27)
HH ^d^ income quintile 2	0.92 (0.52*–*1.62)	1.11 (0.65*–*1.91)	1.26(0.65*–*2.42)
HH ^d^ income quintile 3	0.88 (0.55*–*1.40)	1.14 (0.73*–*1.79)	0.81 (0.48*–*1.36)
HH ^d^ income quintile 4	0.83 (0.51*–*1.35)	0.86 (0.54*–*1.38)	0.84 (0.49*–*1.44)
HH ^d^ income quintile 5	0.71 (0.42*–*1.20)	0.99 (0.59*–*1.64)	0.73 (0.41*–*1.30)
HH ^d^ size	1.00 (0.89*–*1.12)	1.02 (0.91*–*1.14)	1.04 (0.91*–*1.18)
Good self*-*reported health	0.65 (0.44*–*0.95) *	0.65 (0.46*–*0.94) *	0.60 (0.39*–*0.92) **
Great/excellent self-reported health	0.43 (0.29*–*0.64) ***	0.45 (0.31*–*0.66) ***	0.40 (0.26*–*0.61) ***
Health problem	1.16 (0.83*–*1.63)	1.94 (1.40*–*2.69) ***	1.65 (1.14*–*2.40) **
Routine checkup < 12 months	0.55 (0.40*–*0.75) ***	0.84 (0.62*–*1.13)	0.65 (0.46*–*0.91) *
Pseudo R^2^	0.06	0.07	0.08
Significance	<0.001	<0.001	<0.001

+ Each outcome (delayed healthcare due to cost, did not fill prescriptions, and delayed healthcare due to cost and/or did not fill prescription) was modeled individually using multivariate logistic regression. ^a^ Reference groups for the models: non-smoker, female, white race, not married, education level < high school graduate, not working, lowest 20% household income quintile, poor self-reported health, not having a health problem, and not having a routine medical check-up within the past 12 months. ^b^ Table includes all variables adjusted for in the multivariate logistic regression. ^c^ Mean variance inflation factor = 1.36. AOR = adjusted odds ratio; CI = confidence interval; ^d^ HH = household. * *p* ≤ 0.05; ** *p* ≤ 0.01; *** *p* ≤ 0.001.

**Table 3:** Simulated healthcare utilization and compliance between smokers and non-smokers.

	Delayed Healthcare due to Cost (95% CI)	Did not Fill Prescription (95% CI)	Delay cost and/or Did not Fill Prescription (95% CI)
Smoking	68% (66–69%)	59% (57–60%)	78% (78–79%)
Non-smoking	62% (60–63%)	50% (49–52%)	70% (69–71%)
Difference	6%	9%	8%

## Data Availability

The main dataset used for this study is available upon request.
